# Evaluating the Feasibility of a Pilot Exercise Intervention Implemented Within a Residential Rehabilitation Unit for People With Severe Mental Illness: GO HEART: (Group Occupational Health Exercise and Rehabilitation Treatment)

**DOI:** 10.3389/fpsyt.2018.00343

**Published:** 2018-07-27

**Authors:** Nicole H. Korman, Shelukumar Shah, Shuichi Suetani, Karen Kendall, Simon Rosenbaum, Frances Dark, Ketevan Nadareishvili, Dan Siskind

**Affiliations:** ^1^Addiction and Mental Health Services, Metro South Health Services, Brisbane, QLD, Australia; ^2^School of Medicine, University of Queensland, Brisbane, QLD, Australia; ^3^School of Exercise and Nutrition Sciences, Queensland University of Technology, Brisbane, QLD, Australia; ^4^School of Psychiatry, University of New South Wales, Sydney, NSW, Australia

**Keywords:** severe mental illness, exercise, rehabilitation, pilot study, exercise physiology, mental health

## Abstract

**Purpose:** People with severe mental illness are sedentary, have high cardio-metabolic risks and significantly reduced life expectancy. Despite considerable data regarding positive physical and mental health outcomes following exercise interventions, implementation and evaluation of real-world programs is lacking. The primary aim of this study was to assess the feasibility of an exercise intervention implemented by exercise physiology (EP) students within a residential rehabilitation unit for residents with severe mental illness, together with assessment of a range of secondary physical and mental health outcomes pre- and post- the intervention.

**Design:** Single arm, prospective pilot study evaluating outcomes pre- and post- a 10 week intervention.

**Method:** Inactive people with severe mental illness participated in a mixed aerobic and resistance exercise intervention, three times per week for 10 weeks. Data was obtained from a sample of 16 residents with severe mental illness; primary diagnosis schizophrenia (*n* = 12). Primary outcomes were feasibility as assessed using recruitment, retention and participation rates, as well as reasons for withdrawal and amount of exercise achieved. Secondary outcomes included: functional exercise capacity was measured by the 6-min walk test; metabolic data obtained from anthropometric measurements; blood pressure; fasting cholesterol and blood sugar levels; and physical activity levels and mental health as assessed by self- administered questionnaires measured before and after the intervention.

**Results:** Broad level acceptance of the program: high recruitment (81%), retention (77%), and participation (78%) rates were observed. Promising improvements in functional exercise capacity, volume of exercise, and negative symptoms was demonstrated in those who completed.

**Conclusions:** Exercise interventions delivered by EP students in a residential rehabilitation setting for people with SMI are feasible; group setting, supervision and choice for engagement are important considerations. Evaluation of longitudinal, multi site studies, with the addition of dietary interventions within residential rehabilitation units are warranted. Addressing cost feasibility and cost effectiveness of such programs is recommended.

The trial was registered with the Australian New Zealand Clinical Trials Registry (ANZCTR) number, Unique Identifier: ACTRN 12618000478213, http://www.anzctr.org.au Universal trial number (UTN) – U1111-1211-4009.

## Introduction

People living with severe mental illness (SMI) die approximately 15 years earlier than the general population (1), due in part to their increased risk of preventable conditions such as cardiovascular disease (CVD) (2). Modifiable lifestyle risk factors such as low exercise participation, smoking, and poor dietary habits are major contributors to these poor health outcomes, together with the cardio-metabolic burden of antipsychotic medications (3–8). In view of this, there is an urgent need to address modifiable risk factors that contribute to the mortality gap, such as physical inactivity and low cardio-respiratory fitness by developing effective strategies to improve the poor physical health of people with SMI (9, 10).

Individuals with SMI engage in lower amounts of exercise and have lower cardiorespiratory fitness (CRF) compared with the general population (11–13). Exercise protects against chronic disease, and can improve CRF which is inversely related to all-cause mortality (14). An increasing body of evidence also demonstrates a wide range of mental health benefits of exercise for people with SMI, including improved mood, cognition, quality of life, and reduced positive and negative symptoms of schizophrenia. (15–18).

Widespread implementation of exercise interventions within a mental health setting has been limited by a number of factors (19, 20). A prioritization of mental health interventions over physical health interventions by services has limited funds and spending on implementing physical health interventions, such as exercise. Also, mental health professionals often lack the confidence to design and deliver exercise interventions. Furthermore, there has been a lack of evaluation and dissemination of the results of successful real-world exercise interventions within mental health settings to guide other mental health clinicians wishing to develop exercise programs (20–22).

Mental health rehabilitation services support people with more chronic and enduring symptoms than general outpatient services, offer higher support and focus on functional improvements. In Australia, the Community Care Unit (CCU) is a voluntary residential rehabilitation unit for people with SMI, (predominantly living with chronic schizophrenia) in which a focus on holistic recovery is incorporated into the model of service (23) and hence may be an ideal setting for an exercise intervention implementation (24).

Few exercise studies have been conducted within residential rehabilitation services. One study reported difficulties with recruitment and retention (*n* = 14; 36% dropout), but reduced negative symptoms for participants who completed the personal trainer led exercise program (25). Another study conducted in a secure setting reported high adherence (*n* = 8; 0% dropout) to an off-site gym-based aerobic exercise program delivered by an exercise physiologist (EP), but no changes in CRF or endurance (26). A qualitative study (*n* = 6) of participant experiences in a CCU-based exercise intervention delivered by EP's reported that individualization and group dynamics were important aspects of the program (27). To date, studies in these settings have all relied on employed exercise staff to deliver the interventions, which may affect long term feasibility.

The aim of this pilot study was to evaluate a novel model for the provision of exercise interventions within a real-world residential rehabilitation setting. To address the barriers of resource limitations, this model involved senior exercise physiology (EP) students from a university degree program at no direct cost to the service. EP students were mentored in a multidisciplinary mental health team to implement and evaluate a 10-week exercise intervention for people with SMI living in a CCU in Queensland.

## Methods

### Study setting and participants

The study was approved by the Metro South Human Research Ethics Committee (HREC 16/QPAH/042). All subjects provided written informed consent in accordance with the Declaration of Helsinki. Participants were recruited to the single-arm prospective cohort open pilot study from the Coorparoo CCU, a residential rehabilitation unit of the Metro South Addiction and Health Service in Brisbane, Australia. The CCU has capacity for 20 residents with diagnoses of SMI who are admitted for psychosocial rehabilitation. Most residents admitted to the CCU have diagnoses of either schizophrenia, schizoaffective or bipolar disorder as assessed by the CCU Consultant Psychiatrist, using the Diagnostic Statistic Manual of Mental Disorders, Fifth Edition (DSM- 5). Recruitment occurred over a 3-week period prior to the intervention. Inclusion criteria for the study were; (i) current resident at the Coorparoo CCU, (ii) aged 18–65 years, and (iii) able to provide informed consent. Residents were excluded if they; (i) presented with absolute medical contraindication to exercise as determined by a general practitioner, (ii) were considered high risk for aggression or suicide. (As standard clinical practice, all patients of the mental health service are classified as low, medium, or high risk for aggression and suicide by an experienced mental health clinician or consultant psychiatrist, using a standardized risk screening tool) (28), (iii) were considered to have an acutely unstable mental state by the treating psychiatrist; (residents were considered stable if they had not attended the Emergency Department for mental health assessment or been hospitalized in the past 6 months) (iv) were pregnant, or (v) were engaging in an regular exercise or physical activity program outside of the CCU at the time of recruitment. Fourth year EP students assessed potential participants for risks to exercise using the Adult Pre-exercise Screening System (APSS). The APSS provides an evidence-based system for identifying and managing health risks for exercise and it is used to identify people who may have medical conditions, which put them at a higher risk of an adverse event during exercise. The APSS has 3 stages- stages 1 and 2 are self assessed and stage 3 is completed by a fitness professional to identify and stratify risks to exercise, including whether a recommendation of further medical assessment before exercise program is undertaken (29). In this study, participants with identified risks were reviewed by their general practitioner to determine suitability before inclusion. See Supplementary Image 1.

### Intervention

Drawing on earlier research in this population, implementation of this exercise intervention addressed four main considerations (a) assessment of risk level, (b) assessment of cardiorespiratory fitness status, (c) achievement of an achievable intensity level, and (d) strategies to improve motivation and adherence (30).

Intervention duration was 10 weeks and session frequency was three times per week to maximize improvements in fitness (11). The intervention offered engagement in a total volume of moderate intensity exercise that was greater than 90 min per week, above which has been shown to be associated with improvements in mental health outcomes (17). Exercise sessions consisted of a 45-min group circuit in the shared courtyard of the CCU, which comprised a 5-min warm up, and 10-min cool down. The equipment used for the circuit included a recumbent bike, a treadmill, boxing pads and gloves, therabands, step and dumbbells. The circuit stations varied each week and included a mixture of aerobic and resistance exercises. Stations were designed such that they could be modified (regressed/progressed) to suit individual participant capacity and preference. The Borg Category Ratio 10 scale (31) was used to assess rate of perceived exertion (RPE) and to guide intensity of exercise prescription. Initial prescription was aimed at intensity achievable for participants with low initial CRF but progressed to reach moderate intensity by week 3 (11). EP students aimed to maintain an RPE at 2–3 for the first 2 weeks, then increased to a minimum RPE of 4/10 by week 3 and further increased as per individual participant's capacity. Following warm-up, participants rotated through 10–15 stations. The circuit began at intervals of 30 s of exercise interspersed with 30 s of complete rest and progressed to 60 s interval as the participants' functional capacity increased.

Fourth year EP students led the exercise circuit with supervision from an EP Clinical supervisor as part of a practicum placement from their university undergraduate degree program.

This intervention included several evidence based motivational strategies to increase participants' adherence to the program (30);

1. EP students and mental health staff gave validation and encouragement to participants throughout the intervention
2. EP students helped participants set realistic goals, and addressed individual preferences for exercise engagement
3. EP students and the consultant psychiatrist collaborated to identify and problem-solve barriers to participation and liaised regularly with mental health staff in team meetings to enlist their support with this.

Mental health residential rehabilitation staff were prepared for the intervention prior to study onset by provision of information regarding the protocol for the exercise intervention, and information regarding the potential physical and mental health benefits of exercise to people with SMI in emails and discussion in team meetings prior to the intervention beginning.

EP students were provided with an orientation to the facility, education about common mental disorders for residents, aims of the study and their role in delivery of the intervention. They also attended weekly supervision sessions with the consultant psychiatrist to learn more about mental health conditions and strategies to engage people with SMI.

### Primary outcomes

The primary outcome of the current study was feasibility, which was assessed using recruitment, retention and participation rates, reasons for withdrawal and amount of exercise achieved. *Recruitment* was calculated by the proportion of participants recruited to the study from all eligible CCU residents during the 3-week recruitment phase prior to the study onset. *Retention* was calculated by the proportion of those recruited that completed all pre- and post-measures. *Participation* was the proportion of total sessions in the study attended by participants. *Reasons for non-attendance* and *withdrawal* were recorded in the participant's log book together with adverse events.

### Secondary outcomes

Secondary outcomes included indicators of physical and mental health, exercise participation, and exercise motivations.

*Functional exercise capacity* was estimated by the 6-min walk test (6MWT). The 6MWT measures the distance in meters that an individual can walk in a 6-min period on a flat surface. It is a submaximal test that has been shown to be a reliable measure of exercise capacity in people with SMI (32, 33). In the current study, 6MWT was carried out by trained EP students, utilizing the ATS guidelines (34) at baseline and week 10.

*Physical activity* and *sedentary behavior* was assessed using the Simple Physical Activity Questionnaire (SIMPAQ). The SIMPAQ is a 5-item interviewer-administered, self-report clinical tool to assess physical activity and sedentary behavior in the previous week in populations at risk of engaging in high levels of sedentary behavior, such as those with mental illness (35).

At baseline and week 10, *psychotic symptoms* were rated on the Brief Psychiatric Rating Scale (BPRS) through the use of a semi-structured interview guide and an anchored scoring version of the BPRS-A (36, 37). *Negative symptoms* were assessed via the Scale for the Assessment of Negative Symptoms (SANS) (38) using the Interview guide for Assessment of Negative symptoms (IG-SANS) (39).

*Quality of life* was assessed at baseline and at week 10 using the AQol-8D, a reliable and valid instrument, which is particularly suitable when psychosocial elements of health are of importance (40).

The Behavioral Exercise Regulations Questionnaire (BREQ-2) was used to measure *participants' motivations toward exercise* (41). Items on “identified regulation” (finding personal value in reasons for being active) and “intrinsic regulation” (personal enjoyment/liking of exercise) were combined to create a single domain “autonomous regulation,” (defined as the combined mean of intrinsic and identified regulation) as per a previous validation study of the BREQ 2 in patients with SMI (42).

*Metabolic parameters* including fasting lipids, fasting glucose, waist circumference, and blood pressure, resting heart rate, as well as body mass index (BMI) were collected pre-, and post- the 10-week intervention.

### Statistical analyses

Statistical analyses were conducted using SPSS 24 (SPSS 2016). Feasibility measures were summarized with percentages. Normality probability plots and the Shapiro-Wilk statistic were used to check for normality and appropriate nonparametric statistics were applied to outcomes that were not parametric. Normally distributed pre and post outcome measures were tested using a paired *t*-test with significance level of α = 0.05. Mean differences and associated 95% confident intervals (CI) were calculated and Cohen's d statistic was calculated. Non-parametric data was analyzed using medians, interquartile range and Wilcoxon signed rank tests.

## Results

### Baseline characteristics of participants

Baseline characteristics of the study population are shown in Table 1. The mean age of participants was 31 (SD 7.9) of which 3 (30%) were female. The predominant diagnosis was schizophrenia or schizoaffective disorder (90%) (12). The average duration of illness was 10 years (SD 7.3). Half the cohort were taking clozapine, and greater than 80% were either on olanzapine or clozapine, the two most metabolically unfavorable antipsychotics with the highest risk of weight gain (7). More than half of the sample (54%) were current smokers. Of note, even though 23% of participants were screened as having increased risk of adverse event during exercise at baseline, all were cleared to participate in the study after an assessment by a general practitioner.

**Table 1:** Baseline characteristics.

	**Total sample *N* = 13**	**Completers *N* = 10**	**Withdrawals *N* = 3**
**Demographic**			
Female; *n* (%)	4 (30)	3 (30)	1 (33)
Male; *m* (%)	9 (70)	7 (70)	2 (67)
Age (years), mean (SD)	31 (7.9)	32 (8)	27.6 (8)
Duration of illness, years, mean (SD)	10 (7.3)	11.6 (7.3)	5.1 (5.9)
**Psychiatric diagnosis**	***n*** **(%)**	***n*** **(%)**	***n*** **(%)**
Schizophrenia/schizoaffective	12 (92)	9 (90)	3(100)
Bipolar affective disorder	1 (8)	1 (10)	0
Comorbid substance abuse	4 (30)	3 (30)	1 (33)
**Medications**			
Any antipsychotic	12 (92)	10 (100)	2 (66)
Clozapine	7 (54)	7 (70)	0
Olanzapine	4 (30)	3 (30)	1 (33)
**Physical condition**			
Hypertension	1(7)	0 (0)	0 (0)
Diabetes Mellitus	0 (0)	0 (0)	0 (0)
Abnormal lipid profile	4 (30)	4(40)	0 (0)
Current smoker	7 (54)	6 (60)	1 (33)
Physical health problem identified on risk screen	3 (23)	3 (30)	0 (0)

### Recruitment and retention

**Figure 1:**
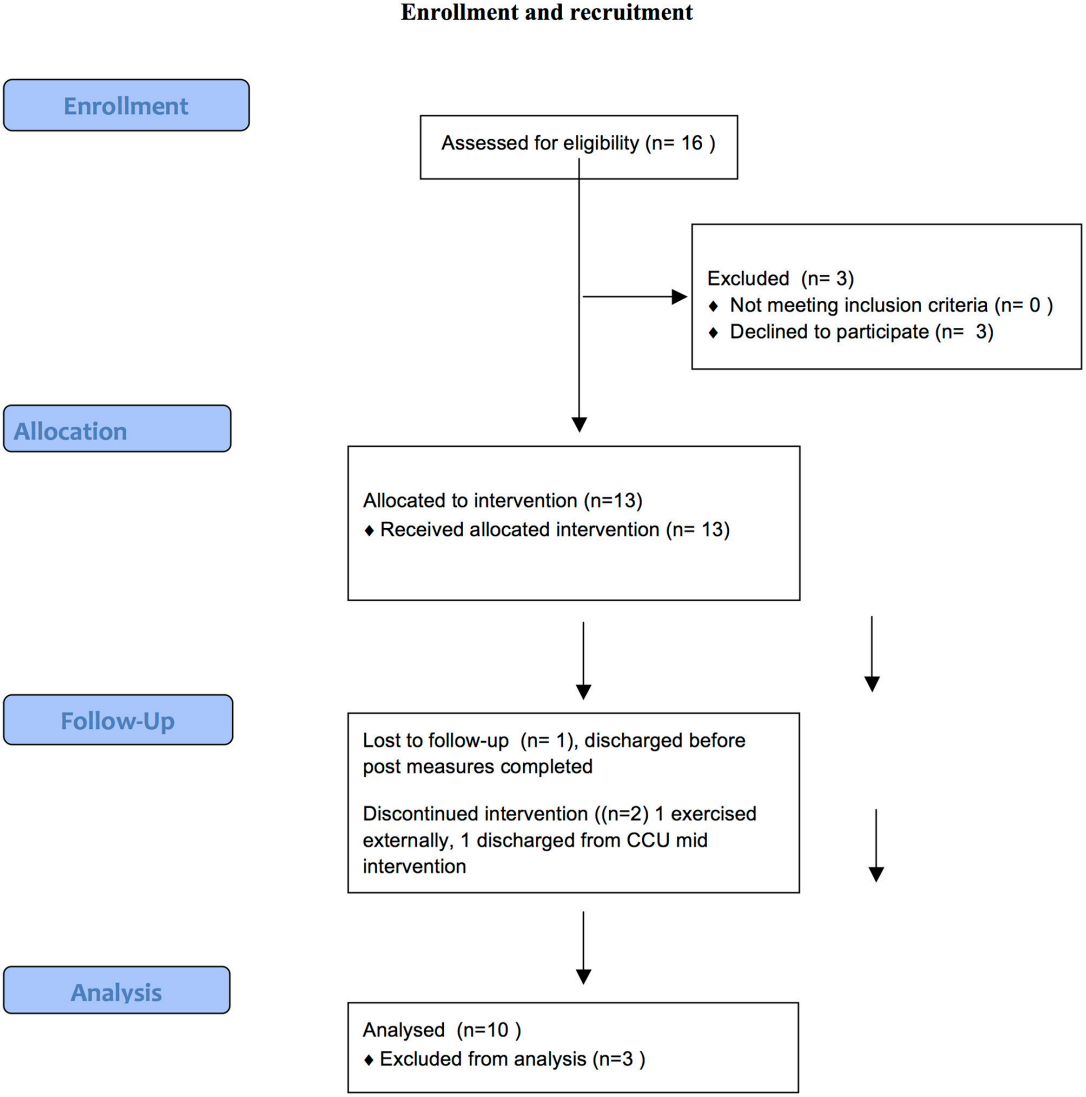
Enrollment and recruitment.

Figure 1 provides a summary of the recruitment and retention process of the current study.

Of 16 eligible residents (81%), consented to participate in the study and completed baseline measures. One resident declined due to lack of interest and two resident's mental state destabilized and hence were not eligible. Ten participants (77%) completed the 10-week study. Of the three who dropped out, one was due to discharge from the service, another preferred to exercise at an external facility, while the third completed the program but was unavailable to complete post measures due to unexpected discharge. Of those who completed the program, mean number of sessions attended was 23.4 (SD 5.34) out of a possible 30, an average attendance rate of 78%.

There were no adverse events during the intervention. Analysis of log books revealed primary reasons for non-attendance were (1) a schedule clash with another appointment (such as GP/ psychologist, blood tests), (2) morning sedation, and (3) feeling unmotivated to attend.

### Pre- and post-measures

Table 2 summarizes the changes in pre- and post-outcome measures after the 10-week exercise intervention. Baseline 6MWT distances for the sample were very low (452 m with SD of 65 m) reflecting reduced initial functional exercise capacity of the participants. Following 10 weeks, the distance achieved on the 6MWT increased significantly by a mean of 76.8 m (95% CI 54, 99), *p* < 0.001 with a large effect size (Cohen's *d* = 0.7). This represented a 16.8% increase on the 6MWT distance. 100% of participants increased their 6MWT distance post the exercise intervention and 80% of participants increased their 6MWT by greater than 50 m.

**Table 2:** Pre- and post-measures.

**(*N* = 10)**	**Baseline mean (SD)**	**Post mean (SD)**	**Mean difference (95% CI)**	**Test statistict (df) or z^b^**	***P*-value**
6MWT distance^1^ (meters)	452 (65)	528 (85)	76.8 (54, 99)	7.78 (9)	<0.001^*^
^np^Self reported exercise in previous week (minutes/week)	60 (52.5)^a^	130 (15)^a^	–	−2.346^b^	0.019^*^
Self-reported sedentary behavior (hours/ day)	7.9 (4.5)	5.8 (2.5)	2.1 (−0.5, 4.7)	−1.82 (9)	0.101
^np^Fasting BSL^2^	5.1 (0.92)^a^	5.15 (0.72)^a^	–	−0.95^b^	*p* > 0.30
Fasting total cholesterol (mmol/L)	4.8 (0.57)	4.6 (0.64)	0.12 (−0.2, 0.44)	−0.83 (9)	*p* > 0.30
Body mass index (weight kg/height m^2^)	29.2 (4.9)	29.4 (4.6)	0.19 (−0.12, −0.9)	0.39 (9)	*p* > 0.30
Abdominal circumference (cm)	100.9 (11.6)	102.8 (12.7)	1.85 (−6.7, 3.01)	0.86 (9)	*p* > 0.30
Systolic blood pressure (mmHg)	134.1 (19.7)	127 (14.5)	7 (−5.8, 19.8)	−1.23 (9)	0.248
Diastolic blood pressure (mmHg)	85 (9.5)	79.3 (8.6)	5.7 (−0.7, 12)	−1.99 (9)	0.078
BPRS^3^	32.4 (8.3)	29 (6.1)	3.4 (0.03, 6.7)	−2.27 (9)	<0.049^*^
SANS^4^	45.2 (11.8)	30.7 (12.7)	14.5 (9.6, 6.8)	6.8 (9)	<0.001^*^
BREQ-2^5^					
^np^Amotivation	0.000 (0.81)^a^	0.000 (0.63)^a^	–	−0.135^b^	*p* > 0.30
^np^External regulation	1.12 (0.56)^a^	1.25 (1.13)^a^	–	−0.949^b^	*p* > 0.30
Introjected regulation	1.7 (1.25)	1.6 (1.08)	0.16 (−0.5, 0.84)	0.56 (9)	*p* > 0.30
Identified regulation	2.45 (1.16)	2.85 (0.94)	0.4 (−1.11, 0.31)	−1.2 (9)	0.235
Intrinsic regulation	2.75 (0.87)	2.5 (0.86)	0.23 (0.06, 0.3)	3.2 (9)	0.01^*^
Autonomous regulation	2.6 (0.8)	2.68 (0.82)	0.03 (−0.9, 0.83)	−0.097 (9)	*p* > 0.30

1 *6MWT, 6 min walk distance*;

2 *BSL, Blood Sugar Level*;

3 *BPRS, Brief Psychiatric Rating Scale*;

4 *SANS, Scale for the Assessment of Negative Symptoms*;

5 *BREQ-2, Behavioral Exercise Regulations Questionnaire*;

* *p < 0.05, significant*;

*np, nonparametric outcomes*.

a *median (Interquartile range) for non-parametric outcomes*.

b *z statistic for Wilcoxon signed rank test for nonparametric outcomes*.

Reported volume of exercise engagement per week (minutes) also increased from pre-intervention (median = 60) to post-intervention (median = 130), *p* = 0.019, with a large effect size (*r* = 0.74). 80% of participants increased their volume of exercise by 60 min or more, 20% reported a 30–60 min reduction compared to baseline. There were no changes in sedentary behavior, metabolic parameters or BMI following 10 weeks of exercise.

In terms of psychiatric symptom measures, both negative symptoms and overall symptoms showed significant reductions. The SANS score decreased by 14.5 (95%CI 9.6, 6.8) *p* < 0.001 with a large effect size (Cohen's *d* = 0.8). All of the participants showed a reduction on their SANS score, with 70% reducing by greater than 10 points, BPRS score decreased by 3.4 (95%CI 0.024, 6.7) *p* < 0.01 post intervention with a small effect size (Cohen's *d* = 0.29).

Participants' attitudes toward exercise as assessed by the BREQ-2 showed significant reduction in intrinsic regulation following the intervention (0.23, CI 0.06, 0.3) *p* = 0.01, with a small effect size (Cohen's *d* = 0.2). However, the change in external motivation domains (amotivation, external regulation and introjected regulation) and autonomous regulation was not significant.

There were no changes seen in the AQoL−8D in any domains or the weighted utility index following the exercise intervention (see Supplementary Table 1).

### Additional observations

This pilot revealed a number of important considerations for conducting exercise interventions in rehabilitation settings. Despite mental health staff being prepared for the exercise intervention prior to implementation, this was found to be insufficient to change the staff culture to allow the exercise intervention to be adequately prioritized from the outset. Initially staff scheduled other appointments or blood tests with participants during exercise session times, reporting that these were more important priorities for residents, which led to reduced participation in exercise. Frequently expressed initial concerns from staff were that if residents exercised they would be too tired to do other therapies. Mental health staff were provided with the opportunity to ask questions and have fears reassured.

Whilst students were given clear initial orientation to the rehabilitation unit and the study, EP students had little prior experience interacting with people with severe mental illness and as such were anxious how to engage them. Students reported feeling uncertain about what to expect from patients and expressed fears about personal safety based on past media exposure. This reflected commonly held attitudes among the general community regarding SMI and the inflated perception of risk of violence (often portrayed in the media) that typically leads to stigma (43). During supervision, the researchers attempted to up skill EP students regarding the accurate evidence of the risk of violence for people with severe mental illness, the consequences of stigma, mental health literacy and the barriers to exercise engagement faced by people recovering from SMI. EP students were also encouraged to have un-structured individual contact with residents of the unit in addition to the exercise intervention (such as playing pool together or games) as this has been reported to be an important aspect of stigma reduction (44).

To improve participation, participants who reported morning sedation were offered a small group make-up session later in the day. Participants who regularly skipped sessions were asked what they would like to do in the program and then preferred elements of the circuit were increased, (i.e., boxing with the EP student) to individualize the program. In the final week of the intervention 3 participants reported loss of interest in some of the circuit elements, which was recorded in logbooks.

## Discussion

### Feasibility

We found high recruitment (81%), retention (78%), and participation (78%) rates, suggesting feasibility of this exercise intervention for patients with SMI in a residential rehabilitation setting. Participation rates were comparable with those reported in two relevant meta-analyses of exercise interventions for people with SMI; (Vancampfort et al. 79–85%, Firth et al. 78.8%). They were also comparable to retention rates reported by the same meta-analyses; (Firth et al. 70–76%, Vancampfort et al. 78%) (17, 30).

For people with SMI, adherence to treatment recommendations is often poor, limiting the recovery of people with severe mental illness (45). Some antipsychotic trials report adherence can be as low as 50 %, with longer term medication adherence rates even lower, (e.g., CATIE trial, 36%) (46, 47). It is worth noting that in our brief pilot study, adherence rates were high and broadly comparable to brief psychological interventions for people with schizophrenia (84%), and depression (83%) (48, 49). This adds to the growing evidence that supports the feasibility of brief exercise interventions for people with SMI (17). However, to achieve a clinically meaning impact on physical health outcomes, engagement with exercise needs to be extended beyond a brief intervention. Future trials of exercise interventions that continue beyond 24 weeks, with follow up periods longer than one year are recommended for people with SMI.

Our recruitment rates (81%) were higher than those seen in Firth's (27.5%), however that study recruited from 4 sites, and hence their recruitment rates may be more generalizable than this single site pilot study (25–27).

Within a residential rehabilitation setting, our participation rates were comparable to Dodd et al's forensic residential rehabilitation study (73%). Firth et al. did not specifically report participation rates, however, did note low adherence as quantified by MET minutes of exercise. These studies differed in terms of flexibility of provision of the supervised exercise sessions, and this may have partially explained the differences in participation results. In our study, EP students were on practicum placement and had the flexibility to offer make-up sessions to participants who missed sessions or preferred a smaller group, hence offering options; Dodd et al. also reported this flexibility. In his residential rehabilitation study, Firth et al. also concluded that tailoring exercise programs to account for participant preference is an important engagement strategy. Given people with SMI frequently experience morning sedation and other medications side effects, offering choice of timing to increase engagement may be a useful strategy when planning exercise programs within residential rehabilitation.

A number of other factors may have contributed to our participation rates. First, the group setting may have allowed participants to encourage each other and provide support; which has been associated with reduced drop-outs in exercise interventions for people with SMI (17). Participants lived within the same residential facility, so exercising in a group may have enhanced social “connectedness,” an important factor for autonomous motivation to engage in exercise for people with SMI (50, 51).

Second, in line with previous exercise intervention studies in this population, supervision from qualified exercise professionals may have improved adherence to the exercise intervention (17, 52). Given the complex barriers that people with SMI face, professional supervision and encouragement is vital when trying to engage this group in exercise; prior studies have shown that access to facilities alone has been unsuccessful in engaging people with SMI in exercise (53, 54).

Third, final year EP students, whilst not fully accredited, generally have significant experience working as an exercise instructor in healthy populations prior to retention of their bachelor degree. In future programs, including a component of mental health training prior to being placed in a mental health service may be a strategy that could assist the EP students to assimilate more comfortably within the service (55). Exercise accreditation bodies are currently improving undergraduate course content to include more comprehensive education about mental illness. In addition, it was observed that EP students expressed attitudes reflecting the misperception of inflated risk of violence by people with SMI that can commonly lead to stigma. Prior stigma-reduction education programs for students have been successful (56). We found that education during supervision and encouraging direct personal contact with residents were important aspects of a student-led intervention to address stigma, and facilitate better integration of EP students into the mental health team.

Fourth, the exercise intervention occurred in the familiar courtyard of the CCU, with no transport or gym costs, hence limiting social and environmental barriers previously reported in the literature, including the anxiety that can be associated with accessing general community facilities (57, 58). For people with SMI living in residential rehabilitation units, who have low exercise confidence and fitness, initial engagement in a familiar onsite location might be preferable preparation for later engagement in mainstream community exercise programs/gyms.

Finally, we found addressing the lack of priority given to physical health by mental health staff was necessary to improve staff engagement, and consequently participants' engagement in the intervention. Unhelpful staff attitudes about physical health has been reported as an important barrier to exercise engagement for people with SMI (57). We addressed this issue by inviting collaborative discussion between mental health staff, EP students and the consultant psychiatrist, to address fears, concerns and myths about people with SMI engaging in exercise interventions, and ensuring comprehensive dissemination of the available evidence about the benefits of physical activity in team meetings and via group email. This may be an important future implementation strategy in services where culture change is important (35, 59). Future implementations could investigate a parallel staff exercise intervention, which may improve staff attitudes toward the use of exercise for people with SMI (60).

### Exercise outcomes within mental health rehabilitation

Consistent with previous findings in SMI (32), our participants' baseline functional exercise capacities, as measured by the 6MWT, were low. However, there was a significant improvement in distance on the 6MWT following our intervention, (mean 70 m). Improvements of greater than 50 m have been shown to be representative of clinically significant change in other chronic disease populations with similarly low baseline 6MWT (61). Increasing functional fitness may have implications for everyday life for people with SMI, a particularly relevant finding in a residential rehabilitation setting in which independent functioning improvements are salient (62).

Our 6MWT improvements were higher than those reported in a secure residential rehabilitation setting, and an outpatient rehabilitation setting; the intensity of exercise in one study was lower than in ours, while the other had a higher mean age of participant (11, 24, 26). The lower baseline functional exercise capacity of our study participants may also have allowed for greater gains in function to be achieved and accounted for the larger change in distance on the 6MWT. Also, as we did not offer a practice test, the impact of learning effects affecting our result cannot be excluded.

We also observed a significant increase in the reported total volume of exercise per week, although participants still did not achieve widely accepted guidelines of 150 min per week of moderate intensity exercise (63). However, given our participants' low baseline exercise participation, the increase we found after a short intervention was promising. Achieving 150 min may be preferable as a long term or “aspirational” goal for people with SMI to prevent early disengagement (64). Whether the increase we found can be sustained or improved upon will require further longitudinal study.

### Other secondary outcomes

Negative symptoms reduced significantly following the intervention. As this study was uncontrolled, this finding may have been due to other factors associated with the rehabilitation program and needs to be interpreted with caution. However prior meta-analyses of randomized controlled trials evaluating exercise interventions in people with SMI, and Firth et al's residential rehabilitation study revealed significant reductions in negative symptoms following exercise interventions (17, 25). Negative symptoms account for a substantial burden on independent functioning and can cause lifelong disability (65). Given the focus on functional improvement for people living in rehabilitation units, exercise interventions that have the potential to impact on negative symptoms may be a worthwhile inclusion in residential rehabilitation programs for people with SMI.

Of note, we found that intrinsic regulation (“I exercise because it's fun”) showed a reduction following the exercise intervention. The EP students deliberately kept circuit elements similar during the 10-week period aiming to increase participants' competence and confidence in performing the circuit stations. This may have led to disinterest in some participants over time, which might provide an explanation. Given the importance of intrinsic motivation in sustaining regular exercise in people with SMI, creating effective exercise interventions that are considered fun/maintain interest over time is relevant and should be a major consideration (66). Consulting with participants to increase popular elements (such as boxing, as observed in this study) in future exercise interventions and offering a variety of physical activities, such as sports, may be worthwhile strategies for consideration. Consumer collaboration in the planning and delivery of exercise interventions, could be worthwhile strategies that would also align with recovery principles of rehabilitation services (20).

Consistent with prior studies of exercise interventions in people with SMI, we found no significant effect on BMI, abdominal circumference or other metabolic outcomes such as fasting blood sugars and lipids. Whilst the brief 10 week intervention may have been too short for change to be seen in metabolic outcomes, the literature reveals inconsistent effects of exercise on metabolic outcomes from exercise alone (11, 17). Whilst fitness improvements without weight loss following an exercise intervention can have a meaningful impact on cardio-metabolic risk reduction, (67), successful weight loss has been demonstrated in people with SMI in lifestyle interventions that included dietary components (24, 68, 69). Residential rehabilitation units typically focus on basic living skill acquisition such as cooking and shopping; as such these units may be an opportune setting to also evaluate dietary interventions that may target changes in metabolic outcomes and the nutrition of people with SMI.

Measures of quality of life have improved following exercise interventions for people with SMI (18). We did not find significant change following this 10 week intervention. Future trials with longer duration may clarify if quality of life measures may change following exercise interventions within residential rehabilitation units.

### Limitations

The pilot study had several limitations. It was a small pilot study and due to the pragmatic nature of the study design, we did not have any blinding process, or a control arm. It is possible that the improvement in functional exercise capacity may have occurred due to unknown external factors, although this is unlikely due to the long term sedentary nature of this population. The improvement in total psychotic and negative symptoms was more at risk of bias as these may have improved due to other aspects of their rehabilitation. Detailed assessment of mood pre and post the intervention and participant and staff acceptability questionnaires would be warranted to understand the broader acceptability of an exercise intervention implemented for people living in residential rehabilitation. Further, we used a measure of self-reported weekly exercise, rather than an objective measure such as accelerometry, thus risking potential inaccuracies and recall bias (70, 71). Of note, whilst the 6MWT has been used to estimate functional exercise capacity in SMI, the correlation between 6MWT and measured CRF in people with SMI has yet to be determined and the minimum clinically important distance has not yet been evaluated in people with SMI. These are both important potential areas of future investigation. Finally, our participants were young and had relatively few physical health co-morbidities, which limits the generalizability of these findings.

### Conclusions

This pilot study revealed the feasibility of a, student-led collaboration in delivering an exercise intervention to a vulnerable, sedentary population of people with SMI, in an era of limited resources within public mental health services. Whilst cost-feasibility was not formally assessed, the intervention was low in cost as it involved EP students on practicum placement, and utilized existing exercise equipment and common space within the residential rehabilitation unit. Further, there was high recruitment, retention and participation rates, and no withdrawals due to injury or adverse events, reflecting feasibility within a residential rehabilitation setting. Elements of the program that may have contributed to feasibility included an on-site, supervised, group program, and offering personalized options for engagement. It may be relevant to other mental health residential rehabilitation units, such as Community Care Units.

### Future directions

Exercise interventions embedded within usual mental health care of a residential rehabilitation unit can be feasible, may lead to improvements in functional exercise capacity and reductions in negative symptoms.

Despite the limitations of pragmatic research, results from this pilot allowed exercise to be incorporated into core rehabilitation programs at three new CCU's, tripling EP student placement opportunities. Given the mortality gap and poor physical health of people with SMI, implementation and evaluation of exercise interventions embedded within MH services is an important next step toward translating evidence into practice.

Larger multi-site studies are warranted to further evaluate the feasibility of implementation of exercise interventions within residential rehabilitation units on a wider scale. The addition of dietary interventions in these settings may be important to have an impact on metabolic outcomes for people with SMI. In addition, studies with longer durations are needed to investigate the sustainability of lifestyle modification programs like this, the long-term benefits of exercise for individuals with SMI, together with the cost-feasibility and cost-effectiveness of such programs (72).

## Data availability statement

The raw data supporting the conclusions of this manuscript will be made available by the authors, without undue reservation, to any qualified researcher.

## Author contributions

NK, KN, DS, FD, KK, SR, and SheS contributed conception and design of the study. KN organized the data base. NK, DS, and SheS performed statistical methods. NK, DS, SheS, and ShuS wrote the first draft of the manuscript. NK, DS, ShuS, SheS, FD, SR, KK, and KN contributed to manuscript revision, read and approved the submitted version.

### Conflict of interest statement

The authors declare that the research was conducted in the absence of any commercial or financial relationships that could be construed as a potential conflict of interest.

## References

[1] Hjorthøj C Stürup A McGrath J Nordentoft M. Years of potential life lost and life expectancy in schizophrenia: a systematic review and meta-analysis. Lancet Psychiatry (2017) 4:295–301. 10.1016/S2215-0366(17)30078-0 28237639

[2] Laursen T Munk-Olsen T Vestergaard M. Life expectancy and cardiovascular mortality in persons with schizophrenia. Curr Opin Psychiatry (2012) 25:83. 10.1097/YCO.0b013e32835035ca 22249081

[3] De Hert M Vancampfort D Correll CU Mercken V Peuskens J Sweers K. Guidelines for screening and monitoring of cardiometabolic risk in schizophrenia: systematic evaluation. Br J Psychiatry (2011) 199:99. 10.1192/bjp.bp.110.084665 21804146

[4] Hahn LA Galletly CA Foley DL Mackinnon A Watts GF Castle DJ. Inadequate fruit and vegetable intake in people with psychosis. Aust N Z J Psychiatry (2014) 48:1025–35. 10.1177/0004867414553950 25296631

[5] Vancampfort D Knapen J Probst M van Winkel R Deckx S Maurissen K. Considering a frame of reference for physical activity research related to the cardiometabolic risk profile in schizophrenia. Psychiatry Res. (2010) 177:271–9. 10.1016/j.psychres.2010.03.011 20406713

[6] Vancampfort D Stubbs B Mitchell A De Hert M Wampers M Ward P. Risk of metabolic syndrome and its components in people with schizophrenia and related psychotic disorders, bipolar disorder and major depressive disorder: a systematic review and meta- analysis. World Psychiatry (2015) 14:339–47. 10.1002/wps.20252 26407790 PMC4592657

[7] Mitchell AJ Vancampfort D Sweers K van Winkel R Yu W De Hert M. Prevalence of metabolic syndrome and metabolic abnormalities in schizophrenia and related disorders—a systematic review and meta-analysis. Schizophr Bull. (2013) 39:306–18. 10.1093/schbul/sbr148 22207632 PMC3576174

[8] Vancampfort D Wampers M Mitchell A Correll C Herdt A Probst M. A meta-analysis of cardio-metabolic abnormalities in drug naïve, first-episode and multi-episode patients with schizophrenia versus general population controls. World Psychiatry (2013) 12:240–50. 10.1002/wps.20069 24096790 PMC3799255

[9] Suetani S Whiteford H McGrath J. An urgent call to address the deadly consequences of serious mental disorders. JAMA Psychiatry (2015) 72:1166–7. 10.1001/jamapsychiatry.2015.1981 26509328

[10] Liu N Daumit G Dua T Aquila R Charlson F Cuijpers P. Excess mortality in persons with severe mental disorders: a multilevel intervention framework and priorities for clinical practice, policy and research agendas. World Psychiatry (2017) 16:30–40. 10.1002/wps.20384 28127922 PMC5269481

[11] Vancampfort D Rosenbaum S Schuch F Ward P Richards J Mugisha J. Cardiorespiratory fitness in severe mental illness: a systematic review and meta-analysis. Sports Med. (2017) 47:343–52. 10.1007/s40279-016-0574-1 27299747

[12] Stubbs B Firth J Berry A Schuch FB Rosenbaum S Gaughran F. How much physical activity do people with schizophrenia engage in? A systematic review, comparative meta-analysis and meta-regression. Schizophr Res. (2016) 176:431–40. 10.1016/j.schres.2016.05.017 27261419

[13] Stubbs B Williams J. How sedentary are people with psychosis? A systematic review and meta-analysis. Schizophr Res. (2016) 171:103–9. 10.1016/j.schres.2016.01.034 26805414

[14] Kaminsky A Arena M Beckie HT Brubaker S Church E Forman AD. The importance of cardiorespiratory fitness in the United States: the need for a national registry: a policy statement from the American Heart Association. Circulation (2013) 127:652–62. 10.1161/CIR.0b013e31827ee100 23295916

[15] Vancampfort D Rosenbaum S Ward P Stubbs B. Exercise improves cardiorespiratory fitness in people with schizophrenia: a systematic review and meta-analysis. Schizophr Res. (2015) 169:453–7. 10.1016/j.schres.2015.09.029 26475214

[16] Firth J Stubbs B Rosenbaum S Vancampfort D Malchow B Schuch F. Aerobic exercise improves cognitive functioning in people with schizophrenia: a systematic review and meta-analysis. Schizophr Bull. (2017) 43:546–56. 10.1093/schbul/sbw115 27521348 PMC5464163

[17] Firth J Cotter J Elliott R French P Yung AR. A systematic review and meta-analysis of exercise interventions in schizophrenia patients. Psychol Med. (2015) 45:1343–61. 10.1017/S0033291714003110 25650668

[18] Battaglia G Alesi M Inguglia M Roccella M Caramazza G Bellafiore M Soccer practice as an add-on treatment in the management of individuals with a diagnosis of schizophrenia. Neuropsychiatric Dis Treat. (2013) 2013:595–603. 10.2147/NDT.S44066 PMC3647379 23662058

[19] Lederman O Suetani S Stanton R Chapman J Korman N Rosenbaum S. Embedding exercise interventions as routine mental health care: implementation strategies in residential, inpatient and community settings. Aust Psychiatry (2017) 25:451–5. 10.1177/1039856217711054 28585448

[20] Rodgers M Dalton J Harden M Street A Parker G Eastwood A Integrated care to address the physical health needs of people with severe mental illness: a rapid review. Health Serv Deliv Res. (2016) 4. 10.3310/hsdr04130 27123505

[21] Suetani S Rosenbaum S Scott JG Curtis J Ward PB. Bridging the gap: what have we done and what more can we do to reduce the burden of avoidable death in people with psychotic illness? Epidemiol Psychiatric Sci. (2016) 25:205. 10.1017/S2045796015001043 26768358 PMC6998739

[22] Vancampfort D Stubbs B Ward P Teasdale S Rosenbaum S. Why moving more should be promoted for severe mental illness. Lancet Psychiatry (2015) 2:295. 10.1016/S2215-0366(15)00099-1 26360074

[23] State of Queensland (Queensland Health) Community Care Unit Model of Service. Mental Health Alcohol and Other Drugs Branch DoH. Fortitude Valley BC, QLD: State of Queensland (Queensland Health).

[24] Daumit GL Dalcin AT Jerome GJ Young DR Charleston J Crum RM. A behavioral weight- loss intervention for persons with serious mental illness in psychiatric rehabilitation centers. Int J Obes. (2010) 35:1114. 10.1038/ijo.2010.224 21042323 PMC3409245

[25] Firth J Carney R Pownall M French P Elliott R Cotter J. Challenges in implementing an exercise intervention within residential psychiatric care: a mixed methods study. Mental Health Phys Activity (2017) 12:141–6. 10.1016/j.mhpa.2017.04.004 28603555 PMC5455809

[26] Dodd K Duffy S Stewart J Impey J Taylor N. A small group aerobic exercise programme that reduces body weight is feasible in adults with severe chronic schizophrenia: a pilot study. Disabil Rehabil. (2011) 33:1222–9. 10.3109/09638288.2010.526162 20950141

[27] Fogarty M Happell B Pinikahana J. The benefits of an exercise program for people with schizophrenia: a pilot study. Psychiatr Rehabil J. (2004) 28:173–6. 10.2975/28.2004.173.176 15605754

[28] Mental Health Directorate QH Queensland Mind Essentials. In Health Q, editor. State of Queensland Brisbane: Queensland Health (2010). p. 96.

[29] Norton KNL Pre-exercise Screening, Guide to the Australian Adult Pre-Exercise Screening System. Exercise and Sports Science Australia, Fitness Australia and Sports Medicine Australia (2011).

[30] Vancampfort D Rosenbaum S Probst M Soundy A Mitchell AJ De Hert M. Promotion of cardiorespiratory fitness in schizophrenia: a clinical overview and meta-analysis. Acta Psychiatr Scand. (2015) 132:131–43. 10.1111/acps.12407 25740655

[31] Borg G. Psychophysical bases of perceived exertion. Med Sci Sports Exerc. (1982) 14:377–81. 10.1249/00005768-198205000-00012 7154893

[32] Bernard P Romain AJ Vancampfort D Baillot A Esseul E Ninot G. Six minutes walk test for individuals with schizophrenia. Disabil Rehabil. (2015) 37:921–7. 10.3109/09638288.2014.948136 25098595

[33] Vancampfort D Probst M Sweers K Maurissen K Knapen J De Hert M. Reliability, minimal detectable changes, practice effects and correlates of the 6-min walk test in patients with schizophrenia. Psychiatry Res. (2011) 187:62–7. 10.1016/j.psychres.2010.11.027 21185084

[34] Borg G ATS Statement: Guidelines for the six-minute walk test. Am J Respir Crit Care Med. (2016) 193:1185 10.1164/rccm.19310erratum 27174486

[35] Rosenbaum S Tiedemann A Stanton R Parker A Waterreus A Curtis J. Implementing evidence-based physical activity interventions for people with mental illness: an Australian perspective. Aust Psychiatry (2016) 24:49–54. 10.1177/1039856215590252 26139698

[36] Crippa JAS Sanches RF Hallak JEC Loureiro SR Zuardi AW. A structured interview guide increases Brief Psychiatric Rating Scale reliability in raters with low clinical experience. Acta Psychiatr Scand. (2001) 103:465–70. 10.1034/j.1600-0447.2001.00185.x 11401662

[37] Woerner MG Mannuzza S Kane JM. Anchoring The BPRS - an aid to improved reliability. Psychopharmacol Bull. (1988) 24:112–7. 3387514

[38] Andreasen N Scale for the assessment of negative symptoms (SANS). Br J Psychiatry (1989) 155(Suppl. 7):53–8. 2695141

[39] Schoemaker J R G M S Interview-Guide-for-IG-SANS. Washington DC. (2010).

[40] Richardson J Iezzi A Khan M Maxwell A. Validity and reliability of the assessment of quality of life (AQoL)-8D multi-attribute utility instrument. the patient: patient-centered outcomes research. (2014) 7:85. 10.1007/s40271-013-0036-x 24271592 PMC3929769

[41] Markland D Tobin V A modification to the behavioural regulation in exercise questionnaire to include an assessment of amotivation. J Sport Exerc Psychol. (2004) 26:191–6. 10.1123/jsep.26.2.191

[42] Vancampfort D De Hert M Vansteenkiste M De Herdt A Scheewe T Soundy A. The importance of self-determined motivation towards physical activity in patients with schizophrenia. Psychiatry Res. (2013) 210:812–8. 10.1016/j.psychres.2013.10.004 24182688

[43] Hocking B A Life Without Stigma: A SANE report. Melbourne, VIC: SANE Australia (2013).

[44] Alliance Q From Discrimmination to Social Inclusion. Queensland, QLT: Queensland Alliance (2009).

[45] Thomas P. The stable patient with schizophrenia–From antipsychotic effectiveness to adherence. Eur Neuropsychopharmacol. (2007) 17:S115–S22. 10.1016/j.euroneuro.2007.02.003 17336766

[46] Martin J Perez V Sacristan M Rodriguez-Artalejo F Martinez C Alvarez E. Meta-analysis of drop-out rates in randomised clinical trials, comparing typical and atypical antipsychotics in the treatment of schizophrenia. Eur Psychiat (2006) 21:11–20. 10.1016/j.eurpsy.2005.09.009 16380237

[47] Lieberman J Stroup TS McEvoy J Swartz M Rosenheck R Perkins D. Effectiveness of antipsychotic drugs in patients with chronic schizophrenia. N Engl J Med. (2005) 353:1209–23. 10.1056/NEJMoa051688 16172203

[48] Binnie J Boden Z Non-attendance at psychological therapy appointments. Mental Health Rev J. (2016) 21:231–48. 10.1108/MHRJ-12-2015-0038

[49] Lincoln T Rief W Westermann S Ziegler M Kesting M Heibach E. Who stays, who benefits? Predicting dropout and change in cognitive behaviour therapy for psychosis. Psychiatry Res. (2014) 216:198–205. 10.1016/j.psychres.2014.02.012 24602992

[50] Vancampfort D De Hert M Stubbs B Ward P Rosenbaum S Soundy A. Negative symptoms are associated with lower autonomous motivation towards physical activity in people with schizophrenia. Compr Psychiatry (2015) 56:128–32. 10.1016/j.comppsych.2014.10.007 25458480

[51] Suetani S Waterreus A Morgan V Foley DL Galletly C Badcock JC. Correlates of physical activity in people living with psychotic illness. Acta Psychiatr Scand. (2016) 134:129–37. 10.1111/acps.12594 27218211

[52] Vancampfort D Rosenbaum S Schuch F Ward P Probst M Stubbs B. Prevalence and predictors of treatment dropout from physical activity interventions in schizophrenia: a meta-analysis. Gen Hosp Psychiatry (2016) 39:15–23. 10.1016/j.genhosppsych.2015.11.008 26719106

[53] Firth J Rosenbaum S Stubbs B Gorczynski P Yung AR Vancampfort D. Motivating factors and barriers towards exercise in severe mental illness: a systematic review and meta-analysis. (2016) 46:2869–81. 10.1017/S0033291716001732 27502153 PMC5080671

[54] Archie S Wilson J Osborne S Hobbs H McNiven J. Pilot study: access to fitness facility and exercise levels in olanzapine-treated patients. Can J Psychiatry (2003) 48:628–32. 10.1177/070674370304800910 14631884

[55] Stanton R Rosenbaum S Lederman O Happell B. Implementation in action: how Australian Exercise Physiologists approach exercise prescription for people with mental illness. J Mental Health (2017) 27:150–6. 10.1080/09638237.2017.1340627 28645230

[56] Economou M Louki E Peppou LE Gramandani C Yotis L Stefanis CN. Fighting psychiatric stigma in the classroom: the impact of an educational intervention on secondary school students' attitudes to schizophrenia. Int J Soc Psych. (2012) 58:544–51. 10.1177/0020764011413678 21828175

[57] Soundy A Stubbs B Probst M Hemmings L Vancampfort D. Barriers to and facilitators of physical activity among persons with schizophrenia: a survey of physical therapists. Psychiatr Serv. (2014) 65:693–6. 10.1176/appi.ps.201300276 24585134

[58] Happell B Scott D Platania-Phung C Nankivell J Nurses' views on physical activity for people with serious mental illness. Mental Health Phys Activity (2012) 5:4–12. 10.1016/j.mhpa.2012.02.005

[59] Fibbins H Ward P Watkins A Curtis J Rosenbaum S. Improving the health of mental health staff through exercise interventions: a systematic review. J Mental Health (2018) 27:184–91. 10.1080/09638237.2018.1437614 29447044

[60] Rosenbaum S Watkins A Ward PB Pearce D Fitzpatrick K Curtis J Psychiatry heal thyself: a lifestyle intervention targeting mental health staff to enhance uptake of lifestyle interventions for people prescribed antipsychotic medication. 2016. p. S619-S.

[61] Redelmeier DA Bayoumi AM Goldstein RS Guyatt GH. Interpreting small differences in functional status: the six minute walk test in chronic lung disease patients. Am J Respir Crit Care Med. (1997) 155:1278. 10.1164/ajrccm.155.4.9105067 9105067

[62] Vancampfort D Guelinckx H Probst M Stubbs B Rosenbaum S Ward P. Health-related quality of life and aerobic fitness in people with schizophrenia. Int J Ment Health Nurs. (2015) 24:394–402. 10.1111/inm.12145 26215311

[63] World Health Organisation. Global Recommendations on Physical Activity for Health. Geneva: World Health Organisation (2010).

[64] Vancampfort D Stubbs B Ward PB Teasdale S Rosenbaum S. Integrating physical activity as medicine in the care of people with severe mental illness. Aust N Z J Psychiatry (2015) 49:681–2. 10.1177/0004867415590831 26041791

[65] Rabinowitz J Levine SZ Garibaldi G Bugarski-Kirola D Berardo CG Kapur S. Negative symptoms have greater impact on functioning than positive symptoms in schizophrenia: analysis of CATIE data. Schizophr Res. (2012) 137:147. 10.1016/j.schres.2012.01.015 22316568

[66] Vancampfort D Stubbs B Venigalla S Probst M. Adopting and maintaining physical activity behaviours in people with severe mental illness: The importance of autonomous motivation. Prev Med. (2015) 81:216–20. 10.1016/j.ypmed.2015.09.006 26386141

[67] Lee D-C Artero E Sui X Blair S Review: Mortality trends in the general population: the importance of cardiorespiratory fitness. J Psychopharmacol. (2010) 24:27 10.1177/1359786810382057 PMC2951585 20923918

[68] Ward P Curtis J Rosenbaum S Watkins A Teasdale S Lederman O Preventing weight gain and increased waist circumference during the first two years after antipsychotic initiation in youth with first-episode psychosis. Eur Psychiatr Assoc. (2016) 33:S114–5. 10.1016/j.eurpsy.2016.01.108

[69] Teasdale SB Ward PB Rosenbaum S Samaras K Stubbs B. Solving a weighty problem: systematic review and meta-analysis of nutrition interventions in severe mental illness. Br J Psychiatry (2017) 210:110. 10.1192/bjp.bp.115.177139 27810893

[70] Firth J Stubbs B Vancampfort D Schuch F Rosenbaum S Ward P. The validity and value of self-reported physical activity and accelerometry in people with schizophrenia: a population-scale study of the UK Biobank. Schizophr Bull. [Epub ahead of print] (2017). 10.1093/schbul/sbx149 29069474 PMC6192495

[71] Soundy A Roskell C Stubbs B Vancampfort D. Selection, use and psychometric properties of physical activity measures to assess individuals with severe mental illness: a narrative synthesis. Arch Psychiatr Nurs. (2014) 28:135–51. 10.1016/j.apnu.2013.12.002 24673789

[72] A-La P McDaid D Weiser P Becker T Kilian R Examining the cost effectiveness of interventions to promote the physical health of people with mental health problems: a systematic review. BMC Public Health (2013) 13:787 10.1186/1471-2458-13-787 23988266 PMC3765875

