# Supplementary material for: Evaluating the Feasibility of a Pilot Exercise Intervention Implemented Within a Residential Rehabilitation Unit for People With Severe Mental Illness: GO HEART: (Group Occupational Health Exercise and Rehabilitation Treatment)
Source: Front Psychiatry. 2018 Jul 27;9:343. doi: 10.3389/fpsyt.2018.00343 (PMC6072846; doi:10.3389/fpsyt.2018.00343)
Supplement: Supplementary file 1 [file Image_1.PDF]

ADULT PRE-EXERCISE SCREENING TOOL

This screening tool does not provide advice on a particular matter, nor does it substitute for advice from an appropriately qualified medical professional. No warranty of safety should result from its use. The screening system in no way guarantees against injury or death. No responsibility or liability whatsoever can be accepted by Exercise and Sports Science Australia, Fitness Australia or Sports Medicine Australia for any loss, damage or injury that may arise from any person acting on any statement or information contained in this tool.

Name: ______________________________

Date of Birth: ______________ Male ☐ Female ☐ Date: ______________

STAGE 1 (COMPULSORY)

AIM: to identify those individuals with a known disease, or signs or symptoms of disease, who may be at a higher risk of an adverse event during physical activity/exercise. This stage is self administered and self evaluated.

Please circle response

1.	Has your doctor ever told you that you have a heart condition or have you ever suffered a stroke?	Yes	No
2.	Do you ever experience unexplained pains in your chest at rest or during physical activity/exercise?	Yes	No
3.	Do you ever feel faint or have spells of dizziness during physical activity/exercise that causes you to lose balance?	Yes	No
4.	Have you had an asthma attack requiring immediate medical attention at any time over the last 12 months?	Yes	No
5.	If you have diabetes (type I or type II) have you had trouble controlling your blood glucose in the last 3 months?	Yes	No
6.	Do you have any diagnosed muscle, bone or joint problems that you have been told could be made worse by participating in physical activity/exercise?	Yes	No
7.	Do you have any other medical condition(s) that may make it dangerous for you to participate in physical activity/exercise?	Yes	No

IF YOU ANSWERED 'YES' to any of the 7 questions, please seek guidance from your GP or appropriate allied health professional prior to undertaking physical activity/exercise

IF YOU ANSWERED 'NO' to all of the 7 questions, and you have no other concerns about your health, you may proceed to undertake light-moderate intensity physical activity/exercise

I believe that to the best of my knowledge, all of the information I have supplied within this tool is correct.

Signature ______________________ Date ______________

Version 1
13/1/2016

EXERCISE INTENSITY GUIDELINES

INTENSITY CATEGORY	HEART RATE MEASURES	PERCEIVED EXERTION MEASURES	DESCRIPTIVE MEASURES
SEDENTARY	< 40% HRmax	Very, very light<br>RPE# < 1	• Activities that usually involve sitting or lying and that have little additional movement and a low energy requirement
LIGHT	40 to <55% HRmax	Very light to light<br>RPE# 1-2	• An aerobic activity that does not cause a noticeable change in breathing rate<br>• An intensity that can be sustained for at least 60 minutes
MODERATE	55 to <70% HRmax	Moderate to somewhat hard<br>RPE# 3-4	• An aerobic activity that is able to be conducted whilst maintaining a conversation uninterrupted<br>• An intensity that may last between 30 and 60 minutes
VIGOROUS	70 to <90% HRmax	Hard<br>RPE# 5-6	• An aerobic activity in which a conversation generally cannot be maintained uninterrupted<br>• An intensity that may last up to about 30 minutes
HIGH	≥ 90% HRmax	Very hard<br>RPE# ≥ 7	• An intensity that generally cannot be sustained for longer than about 10 minutes

# = Borg's Rating of Perceived Exertion (RPE) scale, category scale 0-10

ADULT PRE-EXERCISE SCREENING TOOL

STAGE 2 (OPTIONAL)

Name: ______________________________

Date of Birth: ____________________ Date: ____________________

AIM: To identify those individuals with risk factors or other conditions to assist with appropriate exercise prescription. This stage is to be administered by a qualified exercise professional.

Question	Criteria	RISK FACTORS
1. Age ______<br>Gender ______	≥ 45yrs Males or ≥ 55yrs Females +1 risk factor	
2. Family history of heart disease (eg: stroke, heart attack)<br>Relative / Age / Relative / Age<br>☐ Father ____ ☐ Mother ____<br>☐ Brother ____ ☐ Sister ____<br>☐ Son ____ ☐ Daughter ____	If male < 55yrs = +1 risk factor<br>If female < 65yrs = +1 risk factor<br>Maximum of 1 risk factor for this question	
3. Do you smoke cigarettes on a daily or weekly basis or have you quit smoking in the last 6 months? Yes No<br>If currently smoking, how many per day or week? ______	If yes, (smoke regularly or given up within the past 6 months) = +1 risk factor	
4. Describe your current physical activity/exercise levels: (see table below)	If physical activity level < 150 min/ week = +1 risk factor<br>If physical activity level ≥ 150 min/ week = -1 risk factor<br>(vigorous physical activity/ exercise weighted x 2)	
5. Please state your height (cm) ______<br>weight (kg) ______	BMI = ______________<br>BMI ≥ 30 kg/m² = +1 risk factor	
6. Have you been told that you have high blood pressure? Yes No	If yes, = +1 risk factor	
7. Have you been told that you have high cholesterol? Yes No	If yes, = +1 risk factor	
8. Have you been told that you have high blood sugar? Yes No	If yes, = +1 risk factor	

Question 4 activity levels:

	Sedentary ☐	Light ☐	Moderate ☐	Vigorous ☐
Frequency (sessions per week)				
Duration (minutes per week)				

Note: Refer over page for risk stratification.

STAGE 2 Total Risk Factors = ______

9.	Have you spent time in hospital (including day admission) for any medical condition/illness/injury during the last 12 months? Yes No	If yes, provide details
10.	Are you currently taking a prescribed medication(s) for any medical conditions(s)? Yes No	If yes, what is the medical condition(s)?
11.	Are you pregnant or have you given birth within the last 12 months? Yes No	If yes, provide details. I am ________ months pregnant or postnatal (circle).
12.	Do you have any muscle, bone or joint pain or soreness that is made worse by particular types of activity? Yes No	If yes, provide details

STAGE 3 (OPTIONAL)

AIM: To obtain pre-exercise baseline measurements of other recognised cardiovascular and metabolic risk factors. This stage is to be administered by a qualified exercise professional. (Measures 1, 2 & 3 – minimum qualification, Certificate III in Fitness; Measures 4 and 5 minimum level, Exercise Physiologist*).

	RESULTS		RISK FACTORS
1. BMI (kg/m²)		BMI ≥ 30 kg/m² = +1 risk factor	
2. Waist girth (cm)		Waist > 94 cm for men and > 80 cm for women = +1 risk factor	
3. Resting BP (mmHg)		SBP ≥140 mmHg or DBP ≥90 mmHg = +1 risk factor	
4. Fasting lipid profile*			
Total cholesterol		Total cholesterol ≥ 5.20 mmol/L = +1 risk factor	
HDL		HDL cholesterol >1.55 mmol/L = -1 risk factor<br>HDL cholesterol < 1.00 mmol/L = +1 risk factor	
Triglycerides		Triglycerides ≥ 1.70 mmol/L = +1 risk factor	
LDL		LDL cholesterol ≥ 3.40 mmol/L = +1 risk factor	
5 Fasting blood glucose*		Fasting glucose ≥ 5.50 mmol = +1 risk factor	

STAGE 3 Total Risk Factors = ________

RISK STRATIFICATION

Total stage 2

or

Total stage 3
Plus stage 2 (Q1 - Q4)

≥ 2 RISK FACTORS – MODERATE RISK CLIENTS

Individuals at moderate risk may participate in aerobic physical activity/exercise at a light or moderate intensity (Refer to the exercise intensity table on page 2)

< 2 RISK FACTORS – LOW RISK CLIENTS

Individuals at low risk may participate in aerobic physical activity/exercise up to a vigorous or high intensity (Refer to the exercise intensity table on page 2)

Note: If stage 3 is completed, identified risk factors from stage 2 (Q1-4) and stage 3 should be combined to indicate risk. If there are extreme or multiple risk factors, the exercise professional should use professional judgement to decide whether further medical advice is required.
