# Supplementary material for: Evaluating the Feasibility of a Pilot Exercise Intervention Implemented Within a Residential Rehabilitation Unit for People With Severe Mental Illness: GO HEART: (Group Occupational Health Exercise and Rehabilitation Treatment)
Source: Front Psychiatry. 2018 Jul 27;9:343. doi: 10.3389/fpsyt.2018.00343 (PMC6072846; doi:10.3389/fpsyt.2018.00343)
Supplement: Supplementary file 2 [file Table_1.DOCX]

| **Domain** | Paired differences | Standard Deviation | t statistic | df | Sig, (2 –tailed) |
| --- | --- | --- | --- | --- | --- |
| Independent living | 0.07 | 0.14 | 1.55 | 9 | 0.16 |
| Pain | 0.01 | 0.22 | 0.11 | 9 | 0.92 |
| Senses | -0.04 | 0.14 | -0.84 | 9 | 0.43 |
| Physical | 0.00 | 0.16 | 0.01 | 9 | 0.99 |
| Mental health | 0.03 | 0.15 | 0.69 | 9 | 0.51 |
| Happiness | 0.00 | 0.19 | 0.00 | 9 | 1.00 |
| Coping | -0.08 | 0.19 | -1.42 | 9 | 0.19 |
| Relationships | 0.03 | 0.12 | 0.75 | 9 | 0.47 |
| Self worth | 0.00 | 0.13 | 0.10 | 9 | 0.92 |
| Utility score | 0.01 | 0.19 | 0.10 | 9 | 0.93 |

Supplementary Table 1 – AQoL- 8D
